# Ketamine Treatment for Alcohol Use Disorder: A Systematic Review

**DOI:** 10.7759/cureus.38498

**Published:** 2023-05-03

**Authors:** Michael Kelson, Justin M Burnett, Amy Matthews, Tony Juneja

**Affiliations:** 1 Psychiatry, Hackensack Meridian School of Medicine, Nutley, USA; 2 Medicine, Drexel University College of Medicine, Philadelphia, USA; 3 Psychiatry, Rutgers Robert Wood Johnson Medical School, New Brunswick, USA; 4 Psychiatry, Jersey Shore University Medical Center, Neptune, USA

**Keywords:** systematic review, mental health, addiction, withdrawal, relapse, craving, abstinence, alcohol dependence, alcohol use disorder, ketamine

## Abstract

Alcohol use disorder (AUD) is a chronic, recurrent condition that demonstrates significant heterogeneity in treatment response to first-line agents. Ketamine may have a therapeutic role in substance use disorders; however, research on this topic is limited. The objective of this systematic review is to qualitatively synthesize the current evidence of ketamine treatment for alcohol use disorder and evaluate its efficacy.

A systematic review of Medline, PsycINFO, CINAHL, the Cochrane Library, and Google Scholar was performed to identify completed human studies in English or Spanish (from inception to July 2022) that assess the effectiveness of ketamine therapy for alcohol use disorder. This review was registered on the Open Science Framework. Data were descriptively summarized and presented in tables and tested via narrative synthesis methodology. The risk of bias was measured with Cochrane Collaboration tools and a case series quality assessment tool.

A total of 11 studies with 854 adult patients in three different countries (the USA, the UK, and Russia) were analyzed. Sample sizes ranged from 5 to 211 people. Seven studies included patients with alcohol use disorder, one study focused on heavy drinkers, and three studies elaborated extensively on alcohol withdrawal. The overall proportion of patients achieving abstinence and reduced consumption was most favorable in people receiving combination ketamine and psychotherapy treatment. The results were mixed with respect to relapse, craving, and withdrawal.

Ketamine may be an effective therapeutic modality for people with alcohol use disorders who fail to respond to FDA-approved first-line agents. More robust clinical trials are necessary to provide a more accurate assessment of efficacy, safety profile, and dosing strategies for ketamine utilization in alcohol use disorder.

## Introduction and background

Alcohol use disorder (AUD) is a chronic, recurrent condition characterized by an impaired ability to control alcohol intake despite adverse social, occupational, or health consequences [1,2]. Prior to May 2013, the fourth edition of the Diagnostic and Statistical Manual of Mental Disorders (DSM-IV) classified AUD as two distinct diagnoses: alcohol abuse and alcohol dependence [3]. With the introduction of the DSM-5, these separate disorders were reclassified into a single diagnostic category with mild, moderate, and severe subclassifications [4]. According to the 2020 National Survey on Drug Use and Health (NSDUH), 28.3 million people aged 12 and older were living with alcohol use disorder, which was approximately 8.5% of the United States (U.S.) population [5,6]. The World Health Organization's (WHO) global status report on alcohol and health estimated that 283 million people aged 15 and older had AUD in the year 2016 [7]. It is likely that the prevalence of this condition will continue to rise over the next decade with an uptrend in disease burden [7-9]. And to complicate matters further, public health crises such as the COVID-19 pandemic tend to have serious repercussions for patients living with substance use disorders [10]. Although researchers hypothesize that the level of alcohol use will decline in the short term, they suggest that the long-term consequences of the pandemic will increase alcohol consumption, with a subsequent rise in the number of people meeting diagnostic criteria for alcohol use disorder [10-13].

There are three medications approved by the U.S. Food and Drug Administration (FDA) for the treatment of AUD: acamprosate, disulfiram, and naltrexone [14,15]. Acamprosate is a glutamatergic modulator that seems most effective in decreasing the risk of drinking among already abstinent patients (NNT=12) [14,16]. Disulfiram is an aldehyde dehydrogenase inhibitor that has limited evidence supporting its efficacy in the treatment of AUD [14,15,17]. Naltrexone is a non-selective opioid antagonist, with randomized controlled trials (RCT) supporting its use for decreasing the risk of relapse (NNT=20) or heavy drinking (NNT=12) [14-16]. Although naltrexone and acamprosate are first-line agents recommended to treat AUD, studies have shown significant heterogeneity in treatment response to these medications, leading to variable efficacy rates across patients [18]. Therefore, it is imperative that research and development of more effective options for AUD remain a high priority to advance the field of addiction medicine. One emerging topic in psychiatric research involves the use of psychotomimetic agents for debilitating medical conditions.

Ketamine is an N-methyl-D-aspartate (NMDA) antagonist that was first approved by the FDA in 1970 as an anesthetic agent [19]. Since then, ketamine has long established its role in the operating room, and researchers have continued to investigate its potential benefits in patients with depression, pain syndromes, status epilepticus, and substance use disorders [20]. In 2019, the FDA approved the S-enantiomer of ketamine (esketamine) for patients with treatment-resistant depression (TRD) [21]. Following the success of these studies on TRD with low-dose ketamine infusions, investigators were intrigued by the results and began to evaluate their impact on patients with alcohol use disorder [22-25]. Like depression, AUD is a chronic condition and may thus require repeat infusions to achieve a cumulative and sustained effect on sobriety [1,2]. The objective of this systematic review is to qualitatively synthesize the current evidence of ketamine treatment for alcohol use disorder and evaluate its efficacy.

## Review

Methods

This systematic review followed the Preferred Reporting Items for Systematic Reviews and Meta-Analyses (PRISMA) 2020 guidelines, and the protocol was registered on the Open Science Framework (OSF) Registries (https://osf.io/ert9y) [26,27].

Eligibility Criteria

The Population, Intervention, Comparison, Outcome, and Study (PICOS) framework was utilized to formulate our eligibility criteria for this systematic review. These criteria include: (1) studies reporting alcohol use disorder/alcohol dependence, risky/heavy drinking, or withdrawal symptoms in the adult population; (2) studies that assessed the efficacy of ketamine injections or infusions with or without adjunctive therapeutic modalities; (3) studies with or without comparison groups; (4) studies that mentioned alcohol consumption, abstinence, relapse, cravings, or withdrawal as the outcome measure(s); and (5) studies that were observational (case-control, cohort), experimental (randomized controlled trial), or descriptive (case series) and published in peer-reviewed journals.

Studies were excluded if the primary focus was on a medical condition other than AUD, heavy/harmful drinking, or alcohol withdrawal. Likewise, we excluded studies that focused on ketamine metabolites only or examined pathology unrelated to the treatment of AUD, heavy drinking, or withdrawal symptoms. Furthermore, we excluded all case reports, reviews, abstracts, surveys, dissertations, letters to the editor, conference papers, commentaries, and studies where full text was unavailable.

Search Strategy

An electronic literature search was conducted in July 2022 via five databases: Medline, PsycINFO, CINAHL, the Cochrane Library, and Google Scholar. No restrictions were applied as per the publication date, and only manuscripts written in English or Spanish were reviewed. The comprehensive literature search was performed using Medical Subject Heading (MeSH) terms and keywords. A complete search string of the databases can be found in the supplementary materials of the appendix; however, we employed general search term concepts of AUD and ketamine such as (ketamine OR esketamine) AND (alcohol use disorder OR alcohol dependence OR heavy drinking OR withdrawal) [28]. Additionally, reference lists of included studies were searched for potentially relevant manuscripts.

Study Selection and Data Extraction

The study selection process was conducted in accordance with the Cochrane Collaboration guidelines [29]. All citations were exported to a reference management software (Zotero) [30], and duplicate items were removed. Afterward, the following data was imported into Microsoft Excel 2021 (Microsoft® Corp., Redmond, WA): title; first author; abstract; keywords; publication date; objectives; participant characteristics; location; study design; intervention; control groups; outcome measures; statistical analyses; risk of bias; adverse events. Two investigators (MK and JB) independently screened the titles and abstracts of included studies for eligibility during the first round of screening. Discrepancies or uncertainty between the two reviewers were adjudicated by a third investigator (AM). A full-text review was performed in the second round of screening by two reviewers (MK and JB) independently. Disagreement during this phase was resolved by a third reviewer (AM). In cases where the data was ambiguous or missing, the study authors were contacted to request additional information.

Outcome Measures and Data Analysis

The primary outcomes of concern for this systematic review include alcohol consumption (quantity and frequency), abstinence, relapse, cravings, and withdrawal. Data extracted from the clinical studies were descriptively summarized and presented in tables and text via narrative synthesis methodology. Given the dissimilar magnitude and direction of effect size among the included studies, a high degree of heterogeneity was present with respect to procedural underpinnings and outcome measures. The researchers deemed that a meta-analysis would not be appropriate for this study given the sparse number of clinical trials, their heterogeneous nature, and the risk of bias associated with individual studies [29].

Quality Assessment

Three investigators (MK, JB, and AM) evaluated the methodological quality of the included studies using version 2 of the Cochrane Collaboration Risk-of-Bias tool for randomized trials (RoB 2) [31], the Risk of Bias in Non-Randomized Studies of Interventions (ROBINS-I) [32], and the tool for evaluating the methodological quality of case reports and case series [33]. The revised RoB 2 tool is structured into five domains of bias: bias arising from the randomization process, bias due to deviations from the intended interventions, bias due to missing outcome data, bias in the measurement of the outcome, and bias in the selection of the reported result [31]. This validated tool uses signaling questions and domain-level judgments to classify the risk of bias as low, high, or some concern. These assessments provided the basis for a final risk of bias judgment for the studies evaluated. The ROBINS-I tool consists of seven bias domains: bias due to confounding, bias in the selection of participants for the study, bias in the classification of interventions, bias due to deviations from intended interventions, bias due to missing data, bias in the measurement of outcomes, and bias in the selection of the reported result [32]. Similar to the RoB 2, this is a conceptually rigorous tool that evaluates the risk of bias due to the non-randomization of subjects. This validated tool was used for the observational studies included in this systematic review. The critical appraisal tool created by Murad et al. [33] evaluates the quality of evidence for descriptive studies via signaling questions and four domains of bias: selection, ascertainment, causality, and reporting. This tool employs eight binary responses, which provided the authors with a framework for a final risk of bias judgment (low, moderate, high, unclear).

Results

**Figure 1:**
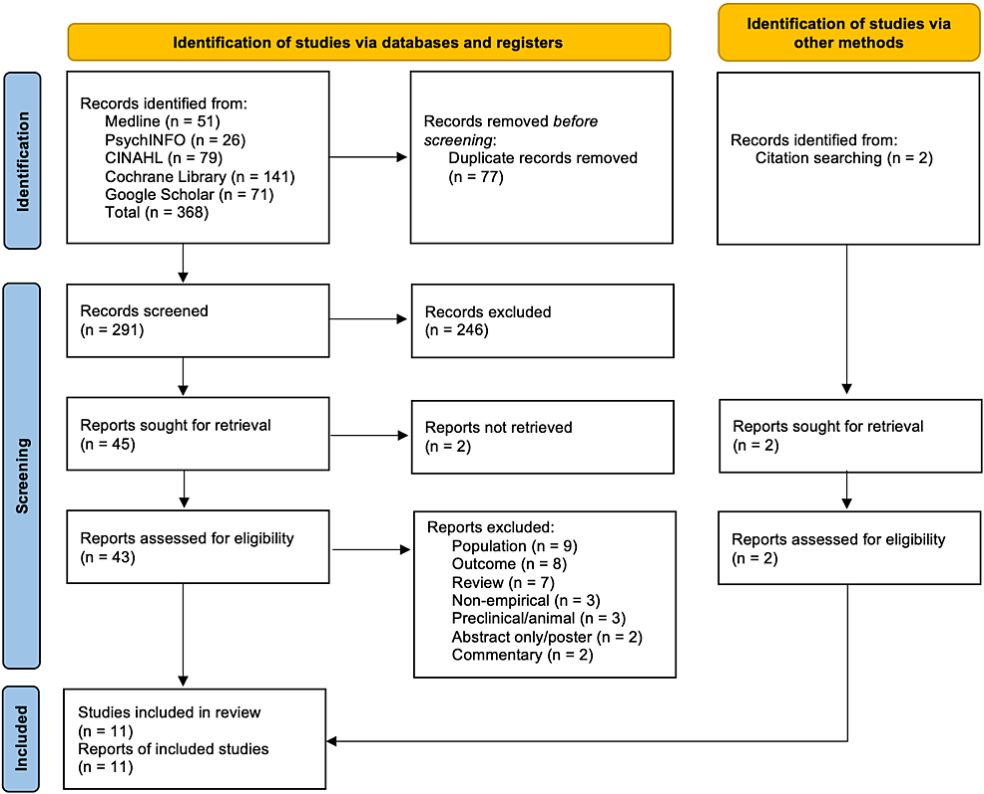
Preferred Reporting Items for Systematic Reviews and Meta-Analyses (PRISMA) flow diagram of study selection. Source: Page MJ, McKenzie JE, Bossuyt PM, et al. The PRISMA 2020 statement: an updated guideline for reporting systematic reviews. BMJ 2021;372:n71.

The initial search strategy yielded a total of 368 records after filters were applied, with two additional references identified through citation searching (Figure 1 for the PRISMA flow diagram). Duplicate items were removed in Zotero and Microsoft Excel 2021, resulting in 291 unique records that were screened for relevance based on title and abstract review. Following the first phase of screening, 45 reports were deemed eligible for full-text appraisal. In total, 11 studies met the inclusion criteria for qualitative synthesis [34-44].

Study Characteristics

The characteristics of the included studies are outlined in chronological order in Table 1. Five of the clinical studies are randomized controlled trials, four utilized a cohort design, and two were conducted as case series. A total of 854 adult patients were analyzed in three different countries (USA, UK, and Russia), and 72.7% of the included studies were published in the last 10 years (n = 8). There is considerable variation in sample size between the studies, ranging from 5 people to 211 participants. Seven studies included patients with alcohol use disorder, whereas one study involved 90 participants who were classified as heavy drinkers and at moderate to high risk of developing AUD. Three of the included studies elaborate extensively on alcohol withdrawal. The duration for most studies ranged from one to three months and only two studies lacked control groups.

**Table 1:** Summary of study characteristics AUD: alcohol use disorder; AUDIT: Alcohol Use Disorders Identification Test; BZD: benzodiazepine; DEX: dexmedetomidine; dL: deciliter; h: hour; IM: intramuscular; IQR: interquartile range; IV: intravenous; kg: kilogram; MDD: major depressive disorder; MET: motivational enhancement therapy; mL: milliliter; mg: milligram; ng: nanogram; RAW: resistant alcohol withdrawal; RCT: randomized controlled trial; SD: standard deviation; UK: United Kingdom; USA: United States of America

Reference	Diagnosis	Sample size/design	Demographic	Intervention	Control	Treatment duration	Follow-up period
Krupitsky et al. [34]	Alcohol dependence	N = 186, RCT	Mean age (intervention) = 33.4 (SD = 1.07), mean age (control) = 38.4 (SD = 0.81), 100% male	IM aethimizol (1.5% 3mL) + IV bemegride (0.5% 10mL) + IM ketamine (3 mg/kg) + psychotherapy	Conventional AUD treatment (aversive emetic therapy, pharmacologic treatment of cravings, psychotherapy)	Unspecified	1-year post-intervention
Krupitsky and Grinenko [35]	Alcohol dependence	N = 211, prospective cohort study	Mean age (intervention) = 36.5 (SD = 7), mean age (control) = 38.4 (SD = 0.81), 100% male	IM aethimizol (1.5% 3 mL) + IV bemegride (0.5% 10 mL) + IM ketamine (2.5 mg/kg) + psychotherapy	Conventional AUD treatment	3 months	Intervention: 1, 2, 3 years post-treatment Control: 1-year post-intervention
Kolp et al. [36]	Alcohol dependence	N ≈ 70, retrospective case series	Age range = 21-64, males & females, ≈50% comorbid psychiatric illness, ≈90% concurrent addictions	IM ketamine (2-3 mg/kg) + psychotherapy (including MET)	No control	1–10 weeks	1-year post-intervention
Wong et al. [37]	Alcohol withdrawal	N = 23, single-group, retrospective, open-label cohort study	Median age = 50 (IQR = 47, 54), 60.9% male, 87% Caucasian, median serum alcohol = 0 mg/dL (IQR = 0, 38.5), 82.6% RAW	IV ketamine (mean initial dose = 0.21 mg/kg/h, SD = 0.11, median infusion dose = 0.20 mg/kg/h, IQR = 0.12, 0.23) ± ketamine loading dose (0.3 mg/kg) + conventional withdrawal treatment	Internal control group (same treatment as the intervention). Note: conventional withdrawal treatment includes BZD ± DEX ± phenobarbital ± propofol ± antipsychotics ± clonidine ± intubation	Mean duration of ketamine infusion = 55.8 hours (SD = 30.5)	12- and 24-hours post-infusion
Pizon et al. [38]	Alcohol withdrawal delirium	N = 63, retrospective, open-label cohort study	Mean age (intervention) = 47 (SD = 9.6), mean age (control) = 53.3 (SD = 12.2), 81% male, median serum alcohol (intervention) = 0, IQR = 0, 43, median serum alcohol (control) = 19, IQR = 0, 224	IV ketamine (0.15-0.3 mg/kg/hr) ± ketamine bolus (0.3 mg/kg) + conventional withdrawal treatment	Conventional withdrawal treatment (BZD ± DEX ± phenobarbital ± propofol ± antipsychotics ± clonidine ± intubation)	Median duration of ketamine infusion = 47 hours (IQR = 35, 71)	Unspecified
Shah et al. [39]	Alcohol withdrawal	N = 30, single-group, retrospective, open-label cohort study	Mean age = 45.6 (SD = 11.7), 82.3% male, mean serum alcohol = 155.4 mg/dL (SD = 154.6)	IV ketamine (median initial dose = 0.75 mg/kg/h, IQR = 0.5, 1.0, mean maximal daily infusion dose = 1.6 mg/kg/h, SD = 0.9) + conventional withdrawal treatment	Internal control group (same treatment as the intervention)	Mean duration of ketamine infusion = 53.7 hours (SD = 39.4)	1, 4-, 8-, 24-, and 48-hours post-infusion
Yoon et al. [40]	AUD & MDD	N = 5, open-label case series	Mean age = 49.2 (SD = 10.7), 80% male, 100% white	Injectable naltrexone (380 mg) + IV ketamine (0.5 mg/kg)	No control	4 weeks	4 weeks post-intervention
Das et al. [41]	Heavy drinkers (moderate-high risk of developing AUD)	N = 90, single-blind RCT	Mean age = 27.5 (SD = 8.1), 61% male, 62% smokers, mean AUDIT score = 9.07 (SD = 1.08)	IV ketamine (350 ng/dL) after alcohol use	Control groups: (1) IV ketamine (350 ng/dL) + no alcohol (2) IV saline (350 ng/dL) after alcohol use	10 days	9 months total: 2 weeks, 3 months, 6 months, 9 months post-intervention
Dakwar et al. [42]; Rothberg et al. [43]	Alcohol dependence	N = 40, double-blind RCT	Mean age = 53 (SD = 9.8), 52.5% female, 70.3% white, 71.8% employed, 90% family history of alcoholism	IV ketamine (0.71 mg/kg) + MET	IV midazolam (0.025 mg/kg) + MET	5 weeks	21 days post-infusion, 6 months post-intervention
Grabski et al. [44]	AUD	N = 96, double-blind RCT	Mean age = 44 (SD = 10.6), 64% male, 42% history of depression, 46% history of anxiety	IV ketamine (0.8 mg/kg) + psychotherapy	IV saline (0.9%) + alcohol education	≈ 1 to 3 months	3 months post-infusion, 6 months post-infusion

A detailed description of outcome measures of interest, methodology for systematic analysis of data, study results, and author conclusions are reported in chronological order in Table 2. The outcome measures of interest for this systematic review include alcohol consumption (quantity and frequency), abstinence, relapse, craving, and withdrawal. Three clinical studies explored drinking behaviors in the participants [41-43], whereas six of eleven studies elaborated on alcohol abstinence [34-36,42-44]. Five studies evaluate cravings [40-44], five studies assess withdrawal [37-39,42,43], and only three reports describe a defined criterion for relapse with the following quantitative measures [42-44]. Alcohol consumption was assessed with the Timeline Followback (TLFB) method, and abstinence was assessed via self-reports from patients, TLFB, urine testing, and alcohol monitoring devices. Cravings were assessed with Likert scales, the Obsessive Compulsive Drinking Scale (OCDS), the Visual Analogue Scale (VAS), and the Alcohol Craving Questionnaire (ACQ-NOW). Withdrawal symptoms were assessed with benzodiazepine (BZD) dose requirements, ICU days, intubations, the Withdrawal Assessment Scale (WAS), the Clinical Institute Withdrawal Assessment for Alcohol (CIWA), and the Motor Activity Assessment Scale (MAAS). Relapse was measured by TLFB and alcohol monitoring devices.

**Table 2:** Summary of outcome measures and findings ACQ-NOW: Alcohol Craving Questionnaire; ANOVA: analysis of variance; AUD: alcohol use disorder; BZD: benzodiazepine; CI: confidence interval; CIWA: Clinical Institute Withdrawal Assessment for Alcohol; CIWA-Ar: Clinical Institute Withdrawal Assessment for Alcohol, revised; g: gram; GABA: gamma-aminobutyric acid; ICU: intensive care unit; KEP: ketamine-enhanced psychotherapy; KET: ketamine; KPT: ketamine psychedelic therapy; MAAS: Motor Activity Assessment Scale; MRM: maladaptive reward memories; NNT: number needed to treat; OCDS: Obsessive Compulsive Drinking Scale; RET: retrieval of maladaptive alcohol memories; SCRAM: Secure Continuous Remote Alcohol Monitor; TLFB: Timeline Followback; UK: United Kingdom; USA: United States of America; VAS: Visual Analogue Scale; WAS: Withdrawal Assessment Scale

Study	Outcomes of interest	Statistical analysis	Results	Author conclusions
Krupitsky et al. [34]	Alcohol abstinence (assessed with monthly self-reported alcohol consumption)	Frequency distribution	Abstinence: 69.8% of patients (N=60/86) in the intervention reported sobriety at 1 year follow-up compared to 24% (N=24/100) in the control group; 24 people (27.9%) in the intervention relapsed and data was not obtained from 2 patients (2.3%); 76 people (76%) in the control group relapsed	The affective contra-attribution method for AUD treatment is more effective than conventional therapy.
Krupitsky and Grinenko [35]	Alcohol abstinence (assessed with monthly self-reported alcohol consumption)	Frequency distribution	Abstinence: 65.8% of people (N=73/111) in the KPT group reported complete sobriety at 1 year follow-up compared to 24% (N=24/100) in the control; 30 people (27%) in the intervention relapsed and data was unobtainable for 8 patients (7.2%); 69 people (69%) in the control relapsed and data was unobtainable from 7 individuals; 40.7% of patients (N=33/81) in the KPT group maintained abstinence at the 2 year follow-up, 46.9% (N=38/81) relapsed, and data was not obtained from 10 people (12.4%); 33.3% of people (N=14/42) in the KPT group maintained sobriety at the 3 year follow-up, 57.2% (N=24/42) relapsed, and data was unobtainable from 4 patients (9.5%)	Ketamine psychedelic therapy appears safe and effective for treatment of alcohol use disorder.
Kolp et al. [36]	Alcohol abstinence (assessed with monthly self-reported alcohol consumption)	Frequency distribution	Abstinence (1-year post-treatment for version 1-5): KEP version #1: ≈25% (N≈4/20); KEP version #2: ≈35% (N≈5/15); KEP version #3: ≈50% (N≈5/10); KEP version #4: ≈60% (N≈6/10); KEP version #5: ≈70% (N≈10/15)	KEP with various adjunctive therapies in a residential setting show promising results for treating alcoholism in the United States.
Wong et al. [37]	Withdrawal symptoms (assessed with BZD dose requirements, WAS)	Wilcoxon rank-sum test; frequency distribution; measures of central tendency and variability	Withdrawal: no significant change in WAS within 6 hours post-ketamine initiation; statistically non-significant change in median BZD requirements of −40.0 (IQR=−106.7, 21.7, p=0.110) and −13.3 mg (IQR=-86.7, 50.0, p=0.330) at 12- and 24-hours post-infusion; mean time to symptom resolution was 5.6 days (SD=1.8)	Ketamine appears to be safe at low doses and may reduce short-term BZD dose requirements in patients with alcohol withdrawal.
Pizon et al. [38]	Withdrawal symptoms (assessed with BZD dose requirements based on WAS >10, ICU days, intubations)	Multivariable linear and logistic regression modeling; t-test; chi-square test; Pearson product-moment correlation coefficient	Withdrawal: significant reduction in the mean benzodiazepine dose in the ketamine group compared to control (mean difference=1,016.6, p=0.02); results favor post-guideline (ketamine) compared to pre-guideline group for mean ICU days (5.7 and 11.2 days respectively, p<0.001) with regression revealing a decrease of 2.83 ICU days (95% CI=-5.58, −0.089, p=0.043); decreased likelihood of intubation with ketamine use (odds ratio=0.14, 95% CI=0.04, 0.49, p<0.01)	Adjunctive ketamine infusions in patients with alcohol withdrawal delirium was associated with reduced GABA agonist requirements, decreased likelihood of intubation, and a shorter length of stay in the ICU.
Shah et al. [39]	Withdrawal symptoms (assessed with BZD dose requirements, CIWA-Ar, MAAS)	Two-sample t-test; frequency distribution; measures of central tendency and variability	Withdrawal: initial symptom control (defined by CIWA-Ar < 20 or if intubated, a MAAS score < 4) achieved by 100% of patients 1-hour post-ketamine infusion; 43% of people (N=13/30) weaned off all infusions within 48 hours of ketamine initiation; significant reduction in lorazepam requirement 1-day post-ketamine initiation (−4 mg/h, p<0.05)	Adjunctive ketamine therapy for severe alcohol withdrawal may enhance symptom control for BZD-refractory patients and reduce infusion requirements.
Yoon et al. [40]	– Craving (assessed with OCDS)	Frequency distribution	Craving: 80% (N=4/5) of people reported improvement in alcohol cravings	Combination treatment with ketamine and naltrexone reduced the urge to drink in most of the study participants.
Das et al. [41]	Alcohol consumption (quantitative drinking days/week, binges/week, and total alcohol use assessed with TLFB) – Craving (assessed with Likert scale)	2 (baseline, post manipulation) x 3 (group) mixed ANOVA (multivariate simple effects analyses); linear mixed models with random intercepts	Consumption: significant reduction in drinking days/week from day 1 (baseline) to day 10 (post-intervention) for the RET + KET group (F(1,89.449)=10.986, p=0.001, $n{_{p}}^{2}$=0.084) and no significant reduction for controls; highly significant reduction in general alcohol use from day 1 to 10 for both the RET + KET group (F(1,89.17)=19.55, p<0.001, $n{_{p}}^{2}$=0.14), equivalent to a decrease of 188 g/week) and the No RET + KET group (F(1,89.17)=6.527, p=0.012, $n{_{p}}^{2}$=0.052), equivalent to a decrease of 109 g/week); significant reduction in binges (>6 drinks/week) from day 1 to 10 was only seen in the RET + KET group (F(1,88.953)=15.821, p<0.001, $n{_{p}}^{2}$=0.116); reductions were seen in weekly alcohol use across all groups from day 10 to the 9 month follow up (F(1,81.684)=12.677, p=0.001); RET + KET group decreased mean weekly consumption at 9 month follow-up from approx. 672 g to approx. 328 g – Craving: significant reduction in only the RET + KET group for urges to drink pre-consumption (F(1,87)=19.703, p<0.001, $n{_{p}}^{2}$=0.185) and post-consumption (F(1,87)=24.46, p<0.001, $n{_{p}}^{2}$=0.219)	Ketamine following alcohol use reduced MRMs, reinforcing effects of alcohol, and long-term drinking levels relative to control groups.
Dakwar et al. [42] and Rothberg et al. [43] (see descriptive analysis below for results specific to this follow-up study)	Alcohol abstinent days (assessed with TLFB; confirmed by urine ethyl glucuronide test; telephone interview 6 months post-trial) – Heavy drinking days (assessed with TLFB) – Time to relapse (assessed with TLFB) – Craving (assessed with VAS) – Withdrawal (assessed with CIWA)	Longitudinal logistic mixed-effects model with a logit link and a random intercept; Kaplan-Meier survival curves; log-rank test	Abstinence: modeled proportion 21-days post-infusion was stable in the ketamine group and decreased substantially in the midazolam group (quadratic effect of time, F=8.21, df=1,797, p=0.004; time-by-treatment interaction, F=25.1, df=1,797, p<0.001; modeled NNT=4); 75% abstinence (N=6/8) at 6 month follow-up in the intervention compared to 27% in the control (N=3/11) – Heavy drinking days: significant reduction with time in the ketamine group (F=12.34, df=1,798, p<0.001); increased with time for midazolam group (odds ratio=1.19, 95% CI=1.14, 1.25, p<0.001) – Time to relapse (defined by first heavy drinking day or dropout): longer time to relapse with ketamine use (x^2^=4.2, p=0.04) compared to midazolam control – Craving: statistically non-significant across groups – Withdrawal: no significant difference across groups	Ketamine increased the probability of abstinence, delayed the time to relapse, and decreased the likelihood of heavy drinking days compared to midazolam.
Grabski et al. [44]	Alcohol abstinent days (%) and relapse: 3 and 6 months after the first infusion (both assessed with TLFB and SCRAM) – Craving (assessed with ACQ-NOW)	Intention-to-treat analysis; sensitivity analyses with multiple imputation method; linear & logistic regression modeling	Abstinence: greater number of days abstinent at 6-month follow-up in ketamine group compared to placebo, pooled across therapy conditions (mean difference=10.1, 95% CI=1.1, 19); results favor ketamine + therapy group compared to saline + education group at 3 month follow-up (mean difference=15.9, 95% CI=3.8, 28.1); non-significant results between ketamine + therapy group and ketamine + education group at 3 month follow-up (mean difference=4.2, 95% CI=−6.7, 15.2); intention-to-treat analysis indicated significant effect for ketamine group compared to placebo at 3 month follow-up (mean difference=9.0, 95% CI=1.3, 16.7) – Relapse (defined by ≥1 heavy drinking day which is >64.8 g/day for men and >52.0 g/day for women): no significant difference across groups within 6 months; positive correlation was found between self-reported drinking days and SCRAM bracelet readings > 0 for participants between the start and end of treatment (r=0.75, p<0.001, 95% CI=0.63, 0.83) – Craving: no significant difference across groups	Ketamine was associated with a significantly greater number of days abstinent from alcohol, with the most favorable results in the ketamine plus therapy group.

Effects on Alcohol Use Disorder

Seven peer-reviewed studies evaluated the efficacy of ketamine for alcohol use disorder [34-36,40,42-44], whereas one study involved heavy drinkers at moderate to high risk of developing AUD [41]. In a randomized controlled trial conducted by Krupitsky et al. [34], the researchers appraised the effectiveness of the "affective contra-attribution" (ACA) method of alcohol dependence treatment, which consists of three stages: (1) introductory psychotherapy, (2) ketamine psychedelic treatment, and (3) group therapy. This model focuses on aversive conditioning to create negative associations with alcohol use as well as psychedelic psychotherapy, which aims to change an individual’s attitude towards alcohol consumption. Participants for this study met eligibility criteria if they received conventional methods for alcoholism treatment (aversive emetic therapy, pharmacologic treatment of cravings, and psychotherapy) for three months, were unable to maintain sobriety for ≥3 months, and experienced definitive alcohol withdrawal symptoms. The 186 patients were randomized into either the intervention group (ACA method of treatment, N=86) or the control group (traditional method for alcoholism treatment, N=100). One year following the treatment regimen, the authors determined ACA efficacy based on the degree of sobriety achieved. Results from this study demonstrate that the contra-attribution procedure yielded stable remission in a large proportion of the alcohol-dependent population studied. The number of alcohol-abstinent patients with full remission was significantly higher in the intervention (69.8%) compared to the control group (24%).

A subsequent prospective cohort study by Krupitsky and Grinenko was performed five years later to analyze the effectiveness of ketamine psychedelic therapy (KPT) versus conventional, standard methods for treating alcohol dependence [35]. The KPT model focuses more on existential and transpersonal psychology, in contrast to Krupitsky’s earlier study, which centered around aversive conditioning. In this observational study, the researchers recruited 211 people with chronic alcohol dependence who could not control their drinking. Three months of therapy were provided at an addiction therapy center, with 111 participants receiving KPT (aethimizol, bemegride, ketamine, and psychotherapy) and 100 patients receiving conventional methods used to treat alcohol use disorder (aversive emetic therapy, pharmacologic treatment of cravings, and psychotherapy). Following three months of inpatient management, the reported abstinence rates were collected for all patients in this study one year after their release. In the KPT group, percent abstinence at one year post-intervention was observed in 65.8% of subjects (N=73/111) as opposed to 24% (N=24/100) in the conventional treatment group. 27% of individuals (N=30) in the KPT group relapsed, whereas 69% (N=69) in the control group struggled with sobriety. Furthermore, follow-up data were obtained at two and three years post-treatment for the intervention group only. At the two-year follow-up interview, 40.7% (N=33/81) of subjects remained abstinent, with a relapse rate of 46.9% (N=38/81). Three years post-intervention, the data report abstinence in 33.3% of subjects (N = 14/42), with a 57.2% (N = 24/42) relapse rate.

Inspired by Krupitsky’s pioneering investigations with ketamine psychedelic therapy, Kolp et al. engaged in the clinical treatment of patients with alcohol use disorder by way of ketamine-enhanced psychotherapy (KEP) [36]. The KEP model replicated the KPT technique in that it relied on the existential and transpersonal effects of ketamine to promote psychotherapeutic benefits. In Krupitsky’s retrospective, informal report of the pilot data collected from patients between 1996 and 1999, approximately 70 people who met DSM-IV criteria for alcohol dependence were treated with KEP [3]. In its first of five variations formulated, Kolp treated ≈20 individuals with individual outpatient psychotherapy weekly for a period of 10 weeks, with one ketamine injection on week seven. Using this first approach yielded alcohol abstinence rates of ≈25% at the one-year follow-up date. Method 2 of Kolp’s model involved the enrollment of 15 patients into a highly structured residential (inpatient) setting for one week’s duration with KEP and 30 hours of psychoeducation, didactic lectures, interactive classes, and growth-oriented encounter groups. The results from this restructured therapeutic plan achieved one-year abstinence rates of ≈35%. In the third variation of Kolp’s treatment approach, only patients with a remote history or infrequent use of psychotomimetic agents or no history of psychedelic substance use were considered eligible for the KEP program. Comparable to the second alteration of his model, Kolp provided ≈10 people with 30 hours of group residential treatment, and the one-year abstinence rate increased to approximately 50%. The fourth adaptation of the KEP model lengthened the inpatient experience from one to two weeks, with 60 hours of psychoeducation, didactic lectures/classes, and encounter groups. The data revealed successful treatment responses for most individuals, with ≈60% (N≈6/10) maintaining sobriety one year post-intervention. In the final development of Kolp’s model, the inpatient component increased from two to three weeks (90 treatment hours) in duration. Kolp kept the same exclusion criteria for patients with an extensive history of psychedelic drug use, and 15 individuals received treatment with a second ketamine injection session. At the one-year follow-up, approximately 70% of participants remained abstinent from alcohol consumption.

In 2019, Yoon et al. published a study that examined the combined effects of naltrexone and ketamine in patients with AUD and comorbid MDD [40]. This eight-week open-label pilot study included patients who were abstinent from alcohol for at least five days prior to the first ketamine infusion. During the four-week ketamine treatment phase, study participants received one injection of naltrexone (380 mg) two to six days prior to the first ketamine dose. Afterward, weekly ketamine infusions (0.5 mg/kg) were administered for a total of four weeks. This combination treatment was associated with reduced depressive symptomatology and alcohol cravings and consumption. As measured by the Montgomery-Asberg Depression Rating Scale, the researchers found that 100% of the subjects experienced antidepressant efficacy by the fourth dose, which was clinically measured by a ≥50% reduction in baseline scores at four hours post-infusion. Moreover, 80% of the participants reported reductions in alcohol cravings and usage as measured by the OCDS.

A single-blind, placebo-controlled, randomized trial by Das et al. explored the use of ketamine in maladaptive reward memories (MRMs), which are conditioned associations between environmental cues (e.g., the taste of beer) and drug reward [41]. In this study, the researchers recruited participants who were primarily beer drinkers, non-treatment seeking, scored >8 on the Alcohol Use Disorders Identification Test (AUDIT), and consumed >240 g/week of alcohol for women or >320 g/week for men. Study participants were randomized into either the intervention (ketamine infusion targeting a plasma concentration of 350 ng/dL over 30 minutes) or placebo groups (ketamine with no alcohol consumption or alcohol use followed by IV saline), with ketamine/saline administration on day 3 of the trial. In the 10 days following the intervention, the researchers analyzed alcohol consumption data using a linear mixed models analysis, which showed a significant reduction in the number of drinking days per week in the ketamine infusion group after retrieval of alcohol-MRMs [F(189.449)=10.986, p=0.001], and no significant reductions in the control groups. Moreover, the intervention demonstrated clinically significant reductions in general alcohol consumption from baseline to post-manipulation [F(1,89.17)=19.55, p<0.001], equaling 188 g of alcohol in a week. Also, the control group receiving ketamine treatment without alcohol consumption saw smaller reductions in alcohol use [F(1,89.17)=6.527, p=0.012], with a decrease of 109 g of ethanol in a week. However, the saline group did not receive any beneficial results as per general alcohol consumption [F(1,89.95)=0.726, p=0.396]. Only the intervention group reported highly significant reductions in weekly binges (>6 drinks per week from baseline to post-intervention). To assess reversion to heavy drinking levels, a follow-up period of nine months was carried out. The data revealed reductions in weekly alcohol consumption across all three groups [F(1,81.684)=12.677, p = 0.001], with no statistically significant differences between the intervention and controls [F(2,81.54)=0.091, p=0.913].

In a randomized, midazolam-controlled pilot trial conducted by Dakwar et al. [42], the researchers observed the effects of a single ketamine infusion combined with motivational enhancement therapy for the treatment of alcohol use disorder. Participants in this study met eligibility criteria if they were <70 years old, met DSM-IV criteria for alcohol dependence and minimum daily (≥4 heavy drinking days over the past 7 days) or weekly use (≥35 drinks per week for men and ≥28 drinks per week for women), and had no other medical or psychiatric illness. Study participants received six motivational enhancement therapy sessions over a five-week period and either a one-time ketamine hydrochloride infusion (0.71 mg/kg) or a midazolam infusion (0.025 mg/kg) during the second week of treatment. Utilizing a longitudinal logistic mixed-effects model with a logit link and a random intercept, results demonstrated that the ketamine infusion group had significant advantages as per the proportion of alcohol-abstinent days, the number of heavy drinking days, as well as the time to relapse. Across the three-week post-infusion follow-up period, 47.1% (N=8/17) of subjects in the ketamine group used alcohol products, in contrast to 59.1% (N = 13/22) in the midazolam group. The control group reported higher rates of heavy drinking days, with a 23.3% difference between the two groups analyzed. Likewise, participants in the ketamine group had a longer time to relapse (x2=4.2, p=0.04) compared to patients in the control group. At the six-month follow-up interview, 75% (N=6/8) of participants in the ketamine group maintained abstinence, as opposed to 27% (N=3/11) of people in the midazolam group.

Rothberg et al. [43] carried out a secondary analysis using the data from this five-week, randomized, double-blind, placebo-controlled trial. The goal of this study was to investigate whether a subset of the psychoactive effects of ketamine, or more specifically, mystical-type experiences, mediates the efficacy of ketamine when combined with motivational enhancement therapy for the treatment of patients with alcohol use disorder. The eight dimensions of mystical experiences embody the eight subscales utilized by the Hood Mysticism Scale (HMS): (1) ego quality, (2) unifying quality, (3) inner subjective quality, (4) temporal/spatial quality, (5) noetic quality, (6) ineffability, (7) positive affect, and (8) religious quality. After the patients in this pilot study received the single ketamine infusion on week 2, mystical experiences were assessed via the HMS, with analyses carried out by a robust regression algorithmic criterion. The results show that patients receiving ketamine scored approximately 38 points higher on the HMS (b=38.149, p<0.05). The researchers observed a negative correlation between the HMS score and the number of heavy drinking days post-infusion (r=−0.466, p<0.05). Also, the HMS was found to be a significant mediator for the negative relationship between the ketamine group and at-risk drinking (Exp(b)=1.045, p<0.05). Moreover, subscale analyses show that HMS scores of ineffability were positively correlated with the proportion of heavy drinking days (r=0.624, p=0.01) whereas scores of positive affect were positively related to alcohol-abstinent days (r=0.613, p<0.05) and the mean number of daily drinks post-infusion (r=0.554, p<0.05).

A double-blind, placebo-controlled phase 2 clinical trial conducted by Grabski et al. studied the effects of ketamine therapy with relapse prevention-based psychological therapy in the treatment of alcohol use disorder [44]. The participants in this study met eligibility criteria if they were 18-65 years old, met DSM-IV/V criteria for moderate-severe AUD, were abstinent from alcohol for at least 24 hours prior to the baseline visit, and had negative urine screens for all drugs except cannabis and benzodiazepines. The 96 participants were randomly assigned to one of four groups: (1) three weekly ketamine infusions (0.8 mg/kg) and psychotherapy, (2) three weekly saline infusions and psychotherapy, (3) three weekly ketamine infusions and alcohol education, and (4) three weekly saline infusions and alcohol education. The infusion weeks were separated by a minimum of one week and a maximum of three weeks, with each session spanning 40 minutes in duration. The therapy sessions (psychotherapy and alcohol education) were provided approximately 24 hours post-infusion. Intention-to-treat analyses were performed to assess alcohol relapse status and the percentage of days abstinent from randomization to the six-month follow-up period. From the data obtained, we can conclude that the intervention (ketamine infusion) was well tolerated and produced favorable results as per the number of days abstinent. At six months of follow-up, using pooled datasets between the treatment and control groups, the intervention produced a significantly greater number of alcohol-abstinent days (mean difference = 10.1, 95% CI = 1.1, 19.0), with the placebo plus psychoeducation group reporting the lowest percentage of days abstinent. In comparison to the saline plus education group at the three-month follow-up, the ketamine plus psychotherapy group achieved the highest rates of abstinence, with a mean difference of 15.9 (95% CI = 3.8, 28.1). However, no significant differences were observed in the odds of relapse (odds ratio = 0.46, 95% CI = 0.12, 1.74).

Effects on Alcohol Withdrawal

Five peer-reviewed studies evaluated the efficacy of ketamine for alcohol withdrawal [37-39,42,43]. However, only three of the included studies are mentioned here, as the pilot trial by Dakwar et al. (and the follow-up study by Rothberg et al.) scantily mention the outcome results for withdrawal symptoms (found in Table 3). In a single-group, retrospective cohort study by Wong et al. [37], the researchers appraised the safety and effectiveness of adjunctive ketamine to benzodiazepine treatment in patients with an alcohol withdrawal syndrome. Participants for this single-center study met eligibility criteria if they were ≥18 years of age and received adjunctive ketamine therapy with a standardized treatment protocol (benzodiazepine ± dexmedetomidine ± phenobarbital ± propofol ± antipsychotics ± clonidine ± intubation) for alcohol withdrawal management. Utilizing a rank-sum test and descriptive statistics, the 23 patients meeting study criteria were analyzed with respect to baseline characteristics, ketamine treatment parameters/outcomes, and withdrawal measures for the patient population. On average, the time from initial treatment of alcohol withdrawal with conventional therapy to ketamine initiation was 33.6 hours (SD=29.1), in contrast to a median of 12.3 hours (IQR=1.5, 42.6) for people with resistant alcohol withdrawal (RAW; defined as a benzodiazepine-equivalent requirement of 40 mg/hour of diazepam for alcohol withdrawal management; benzodiazepine dose equivalents: clonazepam 0.5 mg = alprazolam 1 mg = midazolam 1 mg = lorazepam 1.5 mg = diazepam 10 mg = chlordiazepoxide 25 mg = oxazepam 30 mg). The mean duration of ketamine treatment was 55.8 hours (SD=30.5) with a low median infusion rate of 0.2 mg/kg/hr (IQR=0.12, 0.23) and a total median dose of 9.7 mg (IQR=4.5, 14.2). Ketamine treatment correlated with statistically non-significant reductions in benzodiazepine requirements of −40.0 (IQR=−106.7, 21.7, p=0.110) and -13.3 (IQR=−86.7, 50.0, p=0.330) mg diazepam equivalents at 12- and 24-hours post-ketamine initiation, respectively. Likewise, there were no changes in alcohol withdrawal scores (N=8, WAS=1.0, IQR=−4.5, 2.0) at six hours post-intervention. Altogether, the mean time to resolution of alcohol withdrawal was 5.6 days (SD=1.8) and the mean length of stay in the ICU was 6.3 days (SD=3.0).

**Table 3:** Quality assessment of observational studies (ROBINS-I tool)

Study	Confounding	Selection of participants into the study	Classification of interventions	Deviations from intended interventions	Missing data	Measurement of outcomes	Selection of the reported result	Overall risk of bias
Krupitsky and Grinenko [35]	Serious risk	Serious risk	Low risk	No information	Serious risk	Serious risk	Moderate risk	Serious risk
Wong et al. [37]	Serious risk	Low risk	Moderate risk	Low risk	Low risk	Serious risk	Moderate risk	Serious risk
Pizon et al. [38]	Serious risk	Low risk	Moderate risk	Low risk	Low risk	Low risk	Moderate risk	Moderate risk
Shah et al. [39]	Serious risk	Low risk	Moderate risk	Low risk	Low risk	Serious risk	Moderate risk	Serious risk

A subsequent retrospective cohort study with two of the same researchers was performed several years later to determine if a treatment guideline (established in March 2011) with adjunctive ketamine infusion improves outcomes in patients with severe alcohol withdrawal [38]. In this observational study, the researchers identified a total of 63 patients admitted to the University of Pittsburgh Medical Center ICU who were diagnosed with delirium tremens (DT). The pre-guideline group (January 2008 to March 2011) received conventional symptom-triggered GABA agonist therapy (dose equivalents: 1.5 mg lorazepam = 10 mg diazepam = 3.3 mg phenobarbital), while 35 post-guideline patients were administered conventional treatment and an IV ketamine infusion (0.15-0.3 mg/kg/hr ± 0.3 mg/kg ketamine bolus) until resolution of alcohol withdrawal delirium. The median duration of ketamine infusion lasted 47 hours (IQR=35, 71) with an initial mean infusion dose of 0.24 mg/kg/hr (SD=0.10). The infusion dose during therapy averaged out to 0.19 mg/kg/hr (SD=0.10) and the median total ketamine dose was 825.4 mg (IQR=440, 1456). The data reported fewer mean ICU days in the post-guideline cohort compared to the control (5.7 and 11.2 days, respectively, p<0.001), and linear regression analysis demonstrated a significant decrease in ICU length of stay in the ketamine group by 2.83 days (95% CI=−5.58, −0.089, p=0.043). The intervention displayed significant reductions in the mean benzodiazepine dose in diazepam equivalents required for clinical management (intervention=1508.5 mg, control=2525.1 mg, p=0.02). In comparison to the pre-guideline group, the ketamine plus conventional therapy group was associated with a significant decrease in the likelihood of intubation (odds ratio=0.14, 95% CI=0.04, 0.49, p<0.01). However, no significant differences were observed for the mean length of stay in the hospital between the groups (95% CI=−8.40, 1.08, p=0.13).

A single-center, retrospective cohort study by Shah et al. explored the use of adjunctive ketamine therapy for the reduction of lorazepam infusion requirements and symptom control in patients with benzodiazepine-resistant alcohol withdrawal [39]. In this study, the investigators reviewed electronic medical records of patients receiving ketamine treatment (>1 hour) for alcohol withdrawal in the medical intensive cardiac care unit. People were excluded from this study if ketamine was used for indications other than alcohol withdrawal, ketamine was used without lorazepam infusion, patients did not meet criteria for severe withdrawal (defined by a CIWA-Ar > 20), or patients received dexmedetomidine or propofol during IV ketamine therapy. Prior to ketamine infusion, patients were treated with the intensive care unit's (ICU) severe alcohol withdrawal protocol, which includes lorazepam bolus/infusion, phenobarbital bolus/infusion, IV diazepam, and intubation if necessary. On average, ketamine infusions were initiated 41.4 hours (SD=39.3) after IV lorazepam initiation and continued for an average of 53.7 hours (SD=39.4). The average amount of time patients received lorazepam, phenobarbital, and ketamine infusions totaled 109 hours (SD=64.8). From the data obtained, we can conclude that ketamine infusions were very well tolerated, with notable effects on benzodiazepine requirements and withdrawal symptoms. Results were apparent one-hour post-ketamine therapy, as seen by a decreased requirement in lorazepam infusion rates (from 14.3 to ≈13-13.3) as well as initial symptom control (defined by CIWA-Ar < 20 or if intubated, a MAAS score < 4) in all 30 patients. At 24 hours post-ketamine infusion, the lorazepam infusion requirements decreased by approximately 4 mg/h (p<0.05). In the 48 hours following the intervention, the researchers noted that 43% of people were completely weaned off all infusions. Moreover, 73.3% of patients (N=22/30) required intubation during their hospital stay, however, 16 of these individuals were intubated prior to the start of ketamine therapy. Overall, the mean length of stay in the ICU was 8.2 days (SD=2.4).

Adverse Effects From Ketamine

Six of 11 studies reported adverse effects from the use of ketamine [37-39,42-44], with two of 11 studies explicitly mentioning no observed side effects [34,35]. The cohort study by Wong et al. [37] and subsequent study by Pizon et al. [38] noted oversedation in one patient from ketamine infusion, thus requiring dose adjustment. The study by Shah et al. [39] reported hypertension in 6.7% of patients (N=2/30) within the first hour of ketamine infusion. The authors Dakwar and Rothberg both concluded that there were no serious adverse events associated with the trial drug, with the most common symptoms being sedation (midazolam, N=12; ketamine, N=8) and headache (midazolam, N=4; ketamine, N=6) [42,43]. In the 2022 RCT published by Grabski et al., 53 adverse events (hypertension, tachycardia, euphoria, and low mood) were observed in 20 study participants and rated as definitively (N=7), probably (N=3), or possibly (N=43) related to ketamine infusion [44].

Risk of Bias

The quality assessment is reported in Table 4 for randomized controlled trials, Table 3 for cohort studies, and Table 5 for case series. The risk of bias for most randomized controlled trials was judged to be at either a low risk of bias or raised some concerns in one or more domains. The most frequent areas of concern in RCTs include the measurement of the outcome and the selection of the reported result. 75% of the observational cohort studies (N=3/4) were determined to be at serious risk of bias, with most concerns stemming from confounding and measurement of outcomes. The descriptive case series included in this study were found to be at high risk of bias in most domains. These two studies were deemed to be of critically low quality according to the assessment tool utilized.

**Table 4:** Quality assessment of randomized controlled trials (RoB 2 tool)

Study	Randomization process	Deviations from the intended interventions	Missing outcome data	Measurement of the outcome	Selection of the reported result	Overall risk of bias
Krupitsky et al. [34]	Some concerns	High risk	Low risk	Some concerns	Some concerns	High risk
Das et al. [41]	Low risk	Low risk	Low risk	Some concerns	Low risk	Low risk
Dakwar et al. [42]	Low risk	Low risk	Some concerns	Some concerns	Some concerns	Some concerns
Rothberg et al. [43]	Low risk	Low risk	Some concerns	Some concerns	Some concerns	Some concerns
Grabski et al. [44]	Low risk	Some concerns	Low risk	Some concerns	Low risk	Low risk

**Table 5:** Quality assessment of descriptive studies (tool for evaluating the methodological quality of case reports and case series)

Study	Domain 1: selection	Domain 2: ascertainment	Domain 3: causality	Domain 4: reporting	Overall risk of bias
Kolp et al. [36]	High risk	High risk	High risk	High risk	High risk
Yoon et al. [40]	Unclear (no information)	High risk	High risk	Moderate risk	High risk

Discussion

The findings from this review suggest that ketamine may improve therapeutic success for people struggling with alcohol use disorder. The first studies to ever document ketamine’s effectiveness for achieving sobriety in AUD occurred in the 1990s, when ketamine was considered a widely unacceptable modality of treatment for substance use [34,35]. Shortly thereafter, researchers like Kolp et al. were inspired to pursue research on ketamine psychedelic therapy due to the innovative approach and promising results generated by Krupitsky’s studies [36]. These three studies garnered abstinence rates >60% for participants [34-36], which, according to Krupitsky and peers, is significantly more effective than the optimal treatment response (33%) for alcohol abstinence at that time [45]. However, the results of these preliminary studies were met with several key limitations. Krupitsky’s studies lacked demographic variability, and the included participants sought prior medical treatment for alcohol dependence [34,35]. The case series by Kolp was informally analyzed in a retrospective manner without patient records, lacked control groups or blinding, and had a small sample size (N≈70) [36]. Therefore, it is challenging to draw conclusions and generalize results based on the efficacy of ketamine psychedelic therapy for abstinence in these earlier trials.

Within the past three years, several studies have shown promising results as per improvement in alcohol abstinence days [42-44] and consumption/heavy drinking days [41-43]. The findings from these RCTs imply that there are synergistic actions between psychological therapy and ketamine, which may lead to higher rates of abstinence than with either treatment alone for patients with AUD [42-44]. This suggests that ketamine functions as a "psychoplastogen," thus enhancing neuroplasticity and synaptogenesis and creating a window of time during which behavioral interventions may be more effective [46-48]. Thus, combined treatment with ketamine and psychotherapy can lead to longer-lasting clinical benefits, foster treatment engagement, and promote abstinence. The authors agree that therapy sessions should occur prior to ketamine initiation, during ketamine infusions, and post-intervention to maximize therapeutic efficacy. Moreover, given the results from studies targeting consumption/heavy drinking days [41-43], we deduce that ketamine’s NMDA receptor antagonistic properties have favorable results in decreasing consumption in patients with alcohol dependence, possibly by affecting memory acquisition and reconsolidation mechanisms [41,49-51]. Although the abovementioned studies are of moderate quality evidence as determined by quality assessment tools, there are several limitations that deserve mention. Due to the smaller sample sizes used and rigid enrollment criteria, these studies have limited generalizability. Also, these studies included some participants with previous exposure to the intervention, thus introducing functional unblinding. Additional large-scale RCTs are warranted to increase our understanding of the advantages offered by using ketamine therapy in conjunction with psychotherapy. Likewise, future studies should assess dosing strategies and identifiable biomarkers related to clinical efficacy, as no current studies on AUD explore these topics.

The evidence for improvement in relapse is mixed, with one study and its follow-up analysis suggesting a longer time to relapse with ketamine use [42,43] and a recently published study finding no long-term improvement in the odds of relapse [44]. Additionally, the influence of ketamine treatment on cravings is unclear, with two studies supporting reduced urges to drink [40,41], whereas other studies found no significant differences between the ketamine and control groups [42-44]. Outcome measures for alcohol withdrawal were also mixed, with Dakwar et al. [42] and Rothberg et al. [43] reporting no significant difference across groups. In contrast, three prospective cohort studies show beneficial effects regarding the use of ketamine as an adjunct to benzodiazepines for the management of alcohol withdrawal [37-39]. The limitations of the studies evaluating craving and relapse are found in the preceding paragraph. The cohort studies exploring the use of adjunctive ketamine therapy for withdrawal symptoms provide lower-quality evidence due to a moderate-to-serious risk of confounding, differential misclassification, measurement error, and selective reporting [37-39]. Given that these three studies were carried out retrospectively with the co-administration of numerous pharmacologic agents (in addition to ketamine therapy), it is difficult to draw valid conclusions from the data provided.

Limitations

This systematic review has several fundamental limitations that should be considered. Most of the studies encompassed small sample sizes (N < 100 in nine studies), with the inclusion of two case series. Effective blinding may have been compromised in the trials due to the dissociative and psychogenic properties of ketamine. The authors were unable to conduct a meta-analysis due to heterogeneity among the study design, inclusion criteria, dosing regimen, use of concomitant medications, outcome variables, treatment duration, and follow-up period. Several of the studies were associated with a moderate to high risk of bias due to methodological limitations, primarily concerning measurements of the outcome and the selection of reported results. Moreover, given the strict eligibility criteria of the included studies, the efficacy of ketamine for participants in these studies may not be representative of individuals diagnosed with alcohol use disorder.

## Conclusions

Collectively, these studies reveal that ketamine treatment may lower the probability of alcohol use, reduce heavy drinking days, and increase the proportion of post-infusion abstinent days. These findings are a step in the right direction for the management of alcohol use disorder, a complex condition that currently presents challenges for successful treatment with FDA-approved first-line agents. However, as previously stated, large-scale clinical trials are vital for assessing optimal dosing strategies, identifiable biomarkers related to clinical efficacy, and long-term risks with repeated use. Nevertheless, these studies provide optimism for the future of addiction medicine treatment.

## Appendices

**Table 6:** Medline search strategy (inception to July 2022)

Step	Search criteria	Citations
	((((((((("Ketamine"[Mesh]) OR "Esketamine" [Supplementary Concept]) OR "N-methylketamine" [Supplementary Concept]) OR (ketamine[Title/Abstract])) OR (esketamine[Title/Abstract])) OR (r-ketamine[Title/Abstract])) OR (s-ketamine[Title/Abstract])) OR (ketalar[Title/Abstract])) OR (ketaset[Title/Abstract])) OR (ketanest[Title/Abstract])	22,971
	((((((((((((((((((((("Alcoholism"[MeSh]) OR ("Alcohol Abstinence"[MeSh])) OR ("Drinking Behavior"[MeSh])) OR ("Alcohol Drinking"[MeSh])) OR ("Binge Drinking"[MeSh])) OR ("Alcohol-Related Disorders"[MeSh])) OR ("Alcohol-Induced Disorders"[MeSh])) OR ("Craving"[MeSh])) OR ("Substance Withdrawal Syndrome"[MeSh])) OR ("Alcohol Withdrawal Seizures"[MeSh])) OR ("alcohol related disorder"[Title/Abstract])) OR (alcohol*[Title/Abstract])) OR (drink*[Title/Abstract])) OR ("binge drink*"[Title/Abstract])) OR ("alcohol use disorder"[Title/Abstract])) OR (AUD[Title/Abstract])) OR ("alcohol addict*"[Title/Abstract])) OR ("heavy drink*"[Title/Abstract])) OR ("alcohol intoxicat*"[Title/Abstract])) OR ("alcohol dependen*"[Title/Abstract])) OR ("alcohol consumption"[Title/Abstract])) OR (withdrawal[Title/Abstract])	623,167
	1 AND 2	823
	Limit 3 to humans AND (English OR Spanish)	335
	Limit 4 to clinical trial	51

**Table 7:** PsycINFO search strategy (inception to July 2022)

Step	Search criteria	Citations
1.	(DE "Ketamine" OR DE "Psychedelic Assisted Therapy") OR (ketamine OR r-ketamine OR s-ketamine OR ketalar OR ketaset OR ketanest OR esketamine)	4,440
2.	(DE "Alcohol Abuse" OR DE "Alcoholism" OR DE "Alcohol Drinking Patterns" OR DE "Alcohol Intoxication" OR DE "Alcohol Withdrawal" OR DE "Sobriety" OR DE "Toxic Disorders")) OR (("alcohol-related disorders" OR alcohol* OR drink* OR "binge drink*" OR "alcohol use disorder" OR AUD OR "alcohol addict*" OR "heavy drink*" OR "alcohol intoxicat*" OR "alcohol dependen*" OR "alcohol consumption" or withdrawal)	255,441
3.	1 AND 2	522
4.	Limit 3 to humans AND (English OR Spanish)	339
5.	Limit 4 to clinical trial OR followup study OR retrospective study OR prospective study	26

**Table 8:** CINAHL search strategy (inception to July 2022)

Step	Search criteria	Citations
1.	(MH "Ketamine") OR ketamine OR r-ketamine OR s-ketamine OR ketalar OR ketaset OR ketanest OR esketamine	5,511
2.	(MH "Alcoholism") OR (MH "Alcohol-Related Disorders") OR (MH "Alcohol Drinking") OR (MH "Binge Drinking") OR (MH "Drinking Behavior") OR (MH "Alcohol Abuse") OR (MH "Alcoholism") OR (MH "Alcoholic Intoxication") OR (MH "Craving") OR (MH "Alcohol Withdrawal Syndrome") OR (MH "Alcohol Withdrawal Seizures") OR “alcohol related disorder” OR alcohol* OR drink* OR “binge drink*” OR “alcohol use disorder” OR AUD OR “alcohol addict*” OR “heavy drink*” OR “alcohol intoxicat*” OR “alcohol dependen*” OR “alcohol consumption” OR “withdrawal”	166,646
3.	1 AND 2	231
4.	Limit 3 to English OR Spanish	227
5.	Limit 4 to humans AND research	79

**Table 9:** Cochrane Library search strategy (inception to July 2022)

Step	Search criteria	Citations
1.	(ketamine):ti,ab,kw	6,212
2.	(alcohol):ti,ab,kw	35,179
3.	1 AND 2	141

**Table 10:** Google Scholar search strategy (inception to July 2022)

Step	Search criteria	Citations
1.	allintitle: ketamine	32,700
2.	allintitle: alcohol	484,000
3.	1 AND 2	71

**Table 11:** PRISMA 2020 Checklist Source: Page MJ, McKenzie JE, Bossuyt PM, Boutron I, Hoffmann TC, Mulrow CD, et al. The PRISMA 2020 statement: an updated guideline for reporting systematic reviews. BMJ 2021;372:n71. doi: 10.1136/bmj.n71

Section and Topic	Item #	Checklist item	Reported on page
Title	1	Identify the report as a systematic review.	1
ABSTRACT			
Abstract	2	Provide a structured summary including, as applicable: background; objectives; data sources; study eligibility criteria, participants, and interventions; study appraisal and synthesis methods; results; limitations; conclusions and implications of key findings; systematic review registration number.	1
INTRODUCTION			
Rationale	3	Describe the rationale for the review in the context of existing knowledge.	2
Objectives	4	Provide an explicit statement of the objective(s) or question(s) the review addresses.	2
METHODS			
Eligibility criteria	5	Specify the inclusion and exclusion criteria for the review and how studies were grouped for the syntheses.	3
Information sources	6	Specify all databases, registers, websites, organizations, reference lists, and other sources searched or consulted to identify studies. Specify the date when each source was last searched or consulted.	3
Search strategy	7	Present the full search strategies for all databases, registers, and websites, including any filters and limits used.	3
Selection process	8	Specify the methods used to decide whether a study met the inclusion criteria of the review, including how many reviewers screened each record and each report retrieved, whether they worked independently, and if applicable, details of automation tools used in the process.	3,4
Data collection process	9	Specify the methods used to collect data from reports, including how many reviewers collected data from each report, whether they worked independently, any processes for obtaining or confirming data from study investigators, and if applicable, details of automation tools used in the process.	3,4
Data items	10a	List and define all outcomes for which data were sought. Specify whether all results that were compatible with each outcome domain in each study were sought (e.g., for all measures, time points, analyses), and if not, the methods used to decide which results to collect.	4
	10b	List and define all other variables for which data were sought (e.g., participant and intervention characteristics, funding sources). Describe any assumptions made about any missing or unclear information.	4
Study risk of bias assessment	11	Specify the methods used to assess the risk of bias in the included studies, including details of the tool(s) used, how many reviewers assessed each study and whether they worked independently, and if applicable, details of automation tools used in the process.	4
Effect measures	12	Specify for each outcome the effect measure(s) (e.g., risk ratio, mean difference) used in the synthesis or presentation of results.	4
Synthesis methods	13a	Describe the processes used to decide which studies were eligible for each synthesis (e.g., tabulating the study intervention characteristics and comparing against the planned groups for each synthesis (item #5)).	4
	13b	Describe any methods required to prepare the data for presentation or synthesis, such as handling of missing summary statistics, or data conversions.	4
	13c	Describe any methods used to tabulate or visually display the results of individual studies and syntheses.	4
	13d	Describe any methods used to synthesize results and provide a rationale for the choice(s). If meta-analysis was performed, describe the model(s), method(s) to identify the presence and extent of statistical heterogeneity, and software package(s) used.	4
	13e	Describe any methods used to explore possible causes of heterogeneity among study results (e.g., subgroup analysis, meta-regression).	4
	13f	Describe any sensitivity analyses conducted to assess the robustness of the synthesized results.	N/A
Reporting bias assessment	14	Describe any methods used to assess the risk of bias due to missing results in a synthesis (arising from reporting biases).	4
Certainty assessment	15	Describe any methods used to assess certainty (or confidence) in the body of evidence for an outcome.	4
RESULTS			
Study selection	16a	Describe the results of the search and selection process, from the number of records identified in the search to the number of studies included in the review, ideally using a flow diagram.	4,5
	16b	Cite studies that might appear to meet the inclusion criteria, but which were excluded, and explain why they were excluded.	5
Study characteristics	17	Cite each included study and present its characteristics.	6-9
Risk of bias in studies	18	Present assessments of risk of bias for each included study.	23.24
Results of individual studies	19	For all outcomes, present, for each study: (a) summary statistics for each group (where appropriate) and (b) an effect estimate and its precision (e.g., confidence/credible interval), ideally using structured tables or plots.	9-17
Results of syntheses	20a	For each synthesis, briefly summarise the characteristics and risk of bias among contributing studies.	6-9
	20b	Present results of all statistical syntheses conducted. If meta-analysis was done, present for each the summary estimate and its precision (e.g., confidence/credible interval) and measures of statistical heterogeneity. If comparing groups, describe the direction of the effect.	9-17
	20c	Present results of all investigations of possible causes of heterogeneity among study results.	N/A
	20d	Present results of all sensitivity analyses conducted to assess the robustness of the synthesized results.	N/A
Reporting biases	21	Present assessments of risk of bias due to missing results (arising from reporting biases) for each synthesis assessed.	23,24
Certainty of evidence	22	Present assessments of certainty (or confidence) in the body of evidence for each outcome assessed.	24,25
DISCUSSION			
Discussion	23a	Provide a general interpretation of the results in the context of other evidence.	24,25
	23b	Discuss any limitations of the evidence included in the review.	24,25
	23c	Discuss any limitations of the review processes used.	24,25
	23d	Discuss the implications of the results for practice, policy, and future research.	24,25
OTHER INFORMATION			
Registration and protocol	24a	Provide registration information for the review, including the register name and registration number, or state that the review was not registered.	3
	24b	Indicate where the review protocol can be accessed, or state that a protocol was not prepared.	3
	24c	Describe and explain any amendments to the information provided at registration or in the protocol.	3
Support	25	Describe sources of financial or non-financial support for the review, and the role of the funders or sponsors in the review.	26
Competing interests	26	Declare any competing interests of review authors.	26
Availability of data, code, and other materials	27	The report which of the following are publicly available and where they can be found: template data collection forms; data extracted from included studies; data used for all analyses; analytic code; any other materials used in the review.	N/A
